# Curcumin Improves the Renal Autophagy in Rat Experimental Membranous Nephropathy via Regulating the PI3K/AKT/mTOR and Nrf2/HO-1 Signaling Pathways

**DOI:** 10.1155/2020/7069052

**Published:** 2020-11-01

**Authors:** Qiu Di Tu, Juan Jin, Xiao Hu, Yan Ren, Li Zhao, Qiang He

**Affiliations:** ^1^Department of Nephrology, Zhejiang Provincial People's Hospital, Hangzhou, Zhejiang, China; ^2^People's Hospital of Hangzhou Medical College, Hangzhou, Zhejiang, China; ^3^Key Laboratory of Kidney Disease of Traditional Chinese Medicine in Zhejiang Province, Hangzhou, Zhejiang, China

## Abstract

Membranous nephropathy (MN, also known as membranous glomerulopathy) is one of the many glomerular diseases causing nephrotic syndrome. The literature indicates that autophagy is associated with the homeostasis of podocytes in glomeruli. Curcumin, the main active component in turmeric, has drawn attention for its effective bioactivities against chronic kidney disease. The current study was aimed at assessing the effects of curcumin and exploring the underlying mechanism that mediates autophagy in an animal model of passive Heymann nephritis (PHN) in rats. Passive Heymann nephritis (PHN) was induced in male SD rats by intraperitoneal injection of anti-Fx1A serum. The rats were divided into 3 groups: control (*n* = 10, normal diet), model group (*n* = 10, 0.5% sodium carboxymethylcellulose), and curcumin (*n* = 10, 300 mg/kg/d). The kidney function and oxidative stress indicators were measured using commercial diagnostic kits, and the histomorphology of renal tissues was observed. The number of podocytes was measured by immunohistochemistry. Meanwhile, the autophagosomes in podocyte were analyzed by transmission electron microscopy and the immunofluorescence assay pointing to p62, an autophagic marker. Western blot analyzed the levels of apoptosis, autophagy, PI3K/AKT/mTOR, and Nrf2/HO-1 pathway-associated proteins. The total cholesterol (TC), triglycerides (TG), creatinine (Scr), blood urea nitrogen (BUN), urine volume, and urine albumin of PHN rats were significantly reduced by the administration of curcumin and attenuated renal histomorphological changes in model rats. Meanwhile, curcumin improved the oxidative stress response by decreasing MDA and increasing SOD, GSH, and CAT levels in the kidney of PHN rats. Furthermore, curcumin significantly ameliorated the podocyte loss, along with the fusion, and increased the autophagic vacuoles compared to the PHN control rats. In addition, curcumin downregulated the expression of Bax, Caspase-3, p62, PI3K, p-AKT, and p-mTOR proteins and upregulated the Bcl-2, beclin1, LC3, Nrf2, and HO-1 levels in this animal model. The results provide a scientific basis that curcumin could significantly alleviate the development of MN by inducing autophagy and alleviating renal oxidative stress through the PI3K/AKT/mTOR and Nrf2/HO-1 pathways.

## 1. Introduction

Membranous nephropathy (MN) is a common cause of nephrotic syndrome in adults worldwide, also known as membranous glomerulopathy [1]. It is a glomerular disease, and characteristic changes include proteinuria, the granular deposit of subepithelial immune complexes in the kidney, and diffuse thickening of the glomerular basement membrane (GBM) [2, 3]. MN can be subdivided into idiopathic membranous nephropathy (IMN), originally known as primary MN and secondary MN. As an autoimmune disease, approximately 75% of cases are IMN, which is mainly associated with the antiphospholipase A2 receptor (anti-PLA2R) antibody and thrombospondin type 1 domain-containing 7A (THSD7A) [4, 5], as well as the secondary causes, including infection, autoimmune disease, malignancies, and drugs [3, 6]. There are about 13.28% of primary glomerular diseases in China, mostly concentrated in the elderly with an average of 13% annual growth rate [7], becoming the main type of nephropathy in China.

Podocytes, the glomerular visceral epithelial cells, are highly specialized cells, together with the GBM, and the glomerular endothelial cells, to maintain the renal blood-urine filtration barrier function [8, 9]. During the progress of MN, podocytes are damaged due to the immune complex activating the complement cascade [10]. At present, the injury of podocytes is identified as the central feature of progressive renal diseases [9], including MN [11]. Injury podocytes result in proteinuria and podocyte's loss. Accumulated studies illustrated that the potential mechanisms of podocyte damage related to a wide variety of physiological changes, such as cytokine activation, oxidative stress, inflammation, autophagy, and apoptosis [12, 13]. However, the specific mechanism of renal injury on podocytes has not been clarified.

Autophagy, a self-digestion process, is highly conserved to remove the superfluous and damaged organelles and contributes to regulate the cellular refreshing [14]. Previous studies have suggested that inhibition of autophagy helps in accelerating the pathogenesis of MN, resulting in more severe proteinuria, extensive foot-process effacement, and loss of podocytes. A recent study demonstrated that the abnormally high level of autophagy in podocytes plays a role in reducing podocyte injury [10, 15]. Consistently, it was reported that rapamycin activated podocyte autophagy, consequently reducing the apoptosis of podocyte [15, 16]. Besides, oxidative stress along with the increased production of reactive oxygen species (ROS) impaired the production of antioxidant molecules to cause tissue damage and aggravated nephritis progression [17]. Previously, it has been found that attenuating oxidative stress helps to ameliorate membranous glomerulonephritis by regulating the Nrf2 signaling pathway [18, 19]. Currently, recent research has demonstrated that the PI3K/AKT/mTOR and Nrf2/HO-1signaling pathways play an indispensable role in renal tissue to regulate autophagy [20, 21]. In clinics, the treatment strategies for IMN are mainly on nursing care, immunosuppressive, and glucocorticoid therapy. Worryingly, the main therapeutic approaches are always unsatisfactory and often cause adverse effects or inhibit spontaneous remission of IMN patients. Hence, more research of novel pharmaceuticals is needed to improve the treatment modalities for the MN.

Curcumin, [1,7-bis(4-hydroxy-3-methoxyphenyl)-1,6-heptadiene-3,5-dione], the major polyphenolic active component of turmeric, has been extensively investigated both in vivo and in vitro for six decades [22]. Curcumin has been shown to possess numerous pharmacological renoprotective effects, including antioxidant and anti-inflammatory [20]. Recently, Li et al. demonstrated that the daily administration of curcumin ameliorates glyoxylate-induced calcium oxalate deposition and renal injuries in mice [23]. Furthermore, curcumin has been exhibited to inhibit oxidative stress, inflammation, hyperuricemic in oxonate-induced [24], glycerol-induced [25], and heavy metal-induced [26] of kidney injury animal models. In addition, curcumin has shown significant potential in neuroprotective effects in cerebral ischemia-reperfusion and cognitive impairment by regulating autophagy [27]. Also, in an in vitro study conducted by Ali et al. [28], curcumin significantly improved the renal damage and oxidative stress in adenine-induced chronic kidney disease in rats. However, little is known about the effects of curcumin on autophagy of kidney tissues in the experimentally induced MN.

Based on this, in the current study, we established a passive Heymann nephritis (PHN) model induced by intraperitoneal injection of anti-Fx1A serum. We further measured the various biochemical parameters and histopathological changes involved in renal function, autophagy, and apoptosis levels to further assess the therapeutic effects of curcumin in the PHN rats. Additionally, in this study, the relationship between curcumin and the PI3K/AKT/mTOR along with Nrf2/HO-1 signaling pathways on kidney tissues in PHN rats was investigated.

## 2. Materials and Methods

### 2.1. Animals

Male Wistar rats, Sprague-Dawley (SD) rats, and New Zealand white rabbits were obtained from the Animal Center of Zhejiang Chinese Medical University (Zhejiang, China). All rats were housed under standard specific pathogen-free (SPF) conditions with a 12 h light/dark cycle at 22~24°C and allowed to eat a standard diet and drink ad libitum. In the present study, the rabbits were housed in the clean-grade animal lab. All experiments of the animals were guided and approved by the Animal Care and Use Committee of Zhejiang Chinese Medical University (approval number: ZSLL-2018-044).

### 2.2. PHN Induction and Treatment

The PHN model was used to mimic human MN. The PHN model was prepared with a standard protocol [29]. Briefly, the Fx1A antigen was acquired from the renal cortices of Wistar rats. Then, male New Zealand white rabbits were immunized with Fx1A antigen, and rabbit antiserum was prepared. PHN was induced in 30 male SD rats with body weights of 250~280 g through a single intraperitoneal injection of anti-Fx1A antiserum (6 mL/kg body weight). The SD rats were randomly divided based on the proteinuria level at one week after anti-Fx1 infusion. Then, the selected PHN rats were randomly divided into the following two groups (10 rat/group): the model group and the curcumin group. Besides, ten of the healthy SD rats were selected as normal controls. The control and model group rats received an intragastric administration of 0.5% sodium carboxymethylcellulose (CMC-Na) freshly, and the curcumin group received curcumin (SC-200509, Santa Cruz, USA) at a dosage of 300 mg/kg body weight in CMC-Na solution for 30 days. The dosages of curcumin were referred to as the study of Soetikno et al. [30]. On day 30, the rats were placed individually in metabolic cages (Jeungdo Bio & Plant Co., Seoul, Korea) for 24 h to collect urine. The volume of urine samples was recorded and then centrifuged at 1000 rpm for 10 min, and the supernatants were stored at -20°C until analysis. The next day, rats were all euthanized after being fasted for 12 h with pentobarbital sodium (150 mg/kg), and blood samples were collected from the abdominal aorta and allowed to clot at room temperature, then followed by the centrifugation at 3000 rpm for 15 min to collect serum, and then stored at -80°C for biochemical assays. After that, the kidney of each rat was immediately removed. The right kidney was fixed in 4% paraformaldehyde for histopathological studies, and the other one was stored at -80°C for biochemical analysis.

### 2.3. Serum, Urine, and Kidney Biochemical Analysis

Serum biochemical parameters of albumin (ALB), total protein (TP), total cholesterol (TC), triglycerides (TG), creatinine (Scr), and blood urea nitrogen (BUN) levels were detected using the commercially available kits, ALB (E-EL-R0025, Boyao Biotechnology, Shanghai, China), TP (XY-50067, X-Y Biotechnology, Shanghai, China), and TC (A111-1-1), TG (F001-1-1), Scr (C011-2-1), and BUN (C013-2-1), all from Nanjing Jiancheng Bioengineering Institute, China. The urine ALB (U-ALB) level was measured with a commercial kit, U-ALB (JKSW-E11069), which was purchased from the Jing Kang Biological Engineering Co., Shanghai, China.

The left kidney was excised for 100 mg, then washed in ice-cold saline, and homogenized in ice-cold phosphate-buffered saline with a tissue homogenizer (T10 basic, Germany). The supernatants were centrifuged at 12,000 rpm for 30 min and then used to measure the levels of superoxide dismutase (SOD), glutathione (GSH), Catalase (CAT), and malondialdehyde (MDA) with the enzyme-linked immunosorbent assay (ELISA) kits according to the manufacturer's instructions. The optical density values were measured at 450 nm with a multimode reader (BioTek, Vermont, USA).

### 2.4. Histopathological Analysis

The right kidney samples from rats were fixed in 4% paraformaldehyde for 24 h, dehydrated in graded alcohol, and embedded in paraffin. Then, the paraffin-embedded tissues were cut into 4 *μ*m sections for hematoxylin-eosin (HE), periodic acid-Schiff (PAS), and Masson staining analysis. Collagen deposition and fibrotic lesions were scored semiquantitatively according to a computer-aided point-counting morphometric analysis (MetaMorph, Universal Imaging Co., Downingtown, PA).

### 2.5. Analysis of Podocyte Number in the Glomerulus

Wilms tumor type 1 (WT1), a transcription factor, encodes a zinc finger protein that is associated with the development of renal failure, and located in podocyte nuclei, and is identified as a highly expressed specific marker in mature podocytes [31]. The podocyte number was estimated by staining WT1 in renal samples with immunohistochemistry as described previously [32]. In brief, the 4 *μ*m paraffin-embedded sections of the renal cortex were stained with rabbit polyclonal WT1 antibody (1 : 100; ab180840; Abcam Cambridge, MA) overnight at 4°C. Then, the sections were incubated with an HRP-conjugated secondary antibody (Bioss, Beijing, China) for 1 h at room temperature. Subsequently, the sections were incubated with streptavidin HRP (Bioss, Beijing, China). The number of WT1-positive cells in 30 randomly selected glomeruli was recorded, and the mean value per glomerulus was calculated. The results were displayed as cells/glomerulus.

### 2.6. Transmission Electron Microscopy (TEM) Examination

The renal cortex tissues were corrected and fixed in cold 2.5% glutaraldehyde for 4 h, then washed three times in PBS, and postfixed in 1% osmium tetroxide for 2 h. After being dehydrated, soaked, and embedded, ultrathin sections (70 nm) were prepared and stained using uranyl acetate and lead citrate and then examined and photographed at 80 KV through a transmission electron microscope (H-7500, Hitachi, Japan).

### 2.7. Immunofluorescence Staining

For the immunofluorescence assay, 4 *μ*m thick sections were then washed with PBS and permeabilized with 0.2% Triton X-100 at room temperature for 15 min. After blocking with 5% bovine serum albumin, slides were incubated with primary antibodies against rabbit anti-p62 (1 : 200; ab155686; Abcam, Cambridge, MA) overnight at 4°C. Subsequently, the sections were stained with Alexa Fluor-594-conjugated goat-anti-rabbit IgG H&L secondary antibody (1 : 1000; ab150088; Abcam, Cambridge, MA) for 1 h at 37°C. The nuclei were stained with 4,6-diamidino-2-phenylindole (DAPI, Beyotime, China) for 10 min at 37°C in the dark. Finally, the coverslips were mounted on slides on an antifluorescence quencher, and the images were captured using a fluorescence microscope (DM60008, Leica, Germany) and analyzed with Image-Pro Plus (Medium Cybernetics, Bethesda, MD, USA).

### 2.8. Western Blotting

Kidney cortex tissues (100 mg) were homogenized with RIPA lysis buffer (P0013B, Beyotime, China) containing a complete protease inhibitor mixture (P1005, Beyotime, China) and then centrifuged at 12,000 rpm for 20 min at 4°C. After that, the protein concentration was quantified by the BCA protein assay kit (Beyotime, China). Next, 50 *μ*g of total lysate from kidney tissue was subjected to be separated in 8%~10% SDS-PAGE electrophoretic gel and transferred to PVDF membrane (Bio-Rad, USA) for 1.5 h. The membranes were blocked in 5% nonfat milk at room temperature for 1 h and then incubated with primary antibodies specific for rabbit monoclonal anti-beclin1 (1 : 1000; ab210498), rabbit polyclonal anti-LC3B (1 : 3000; ab210498), rabbit monoclonal anti-Bcl-2 (1 : 1000; ab32124), anti-Bax (1 : 2000; ab32503), anti-Caspase-3 (1 : 2000; ab184787), rabbit monoclonal anti-GAPDH (1 : 5000; ab181602), and anti-*β*-actin (1 : 5000; ab179467), all from Abcam Biotechnology, or mouse monoclonal anti-PI3K (1 : 200; sc-1637), anti-p-AKT (1 : 200; sc-1637), and anti-p-mTOR (1 : 200; sc-293089), all from Santa Cruz Biotechnology, or rabbit monoclonal anti-Nrf2 (1 : 1000; #12721) and anti-HO-1 (1 : 1000; #82206), both from Cell Signaling Technology at 4°C overnight. After adequate washing, the membranes were incubated with goat anti-rabbit IgG/HRP antibody (1 : 3000; SE134, Solarbio, USA) or goat anti-mouse IgG/HRP antibody (1 : 3000; SE131, Solarbio, USA), and blots were visualized with an enhanced fluorochemiluminescent system (ECL, Beyotime, China). Finally, the relative intensity of the above-mentioned targets normalized to GAPDH was analyzed by the ImageJ software (National Institutes of Health, USA).

### 2.9. Statistical Analysis

Data are expressed as the mean ± standard deviation (SD). All statistical comparisons were analyzed using one-way ANOVA, and the *t*-test was used for the comparison between the two groups using SPSS version 22.0 (SPSS, Inc., IL, USA). *P* < 0.05 was considered statistically significant.

## 3. Results

### 3.1. Effects of Curcumin on Renal Function and Oxidative Stress Response in PHN Rats

As shown in Table 1, the serum of ALB and TP levels in the model group showed a significant decrease, and the TC, TG, SCr, BUN, and urine volume along with urine albumin of rats were markedly increased as compared with that in the control group. Compared with the model group, the ALB and TP in the curcumin group were higher than that in the model, and TC, TG, SCr, BUN, urine volume, and urine albumin were significantly decreased with curcumin treatment. Numerous lines of evidence suggest that oxidative stress is associated with the process of MN [17]. In the present study, we investigated the effects of curcumin in the PHN rat model. As shown in Table 2, there were significantly increased SOD, GSH, and CAT content as well as decreased MDA level in the kidneys of rats in the curcumin group compared to those in the model group. Together, these results indicated that curcumin ameliorates renal function and against the oxidative stress reaction in PHN rats.

### 3.2. Effects of Curcumin on Renal Pathology in PHN Rats

**Figure 1:**
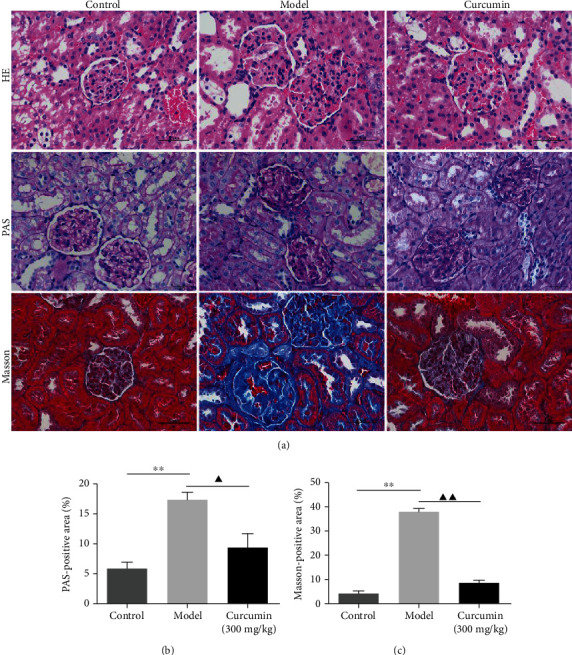
Curcumin improves the morphological changes in renal tissues in PHN rats. (a) Renal tissues were investigated by staining with H&E (scale bars 50 *μ*m), PAS (scale bars 20 *μ*m), and Masson (scale bars 50 *μ*m) and photographed by a light microscope (400x magnification). (b) Semiquantitative analysis of the PAS-positive area was measured by Image-Pro Plus 6.0. (c) Semiquantitative analysis of Masson-positive area was measured by Image-Pro Plus 6.0. ^∗∗^*P* < 0.01, the model group vs. the control group, ^▲^*P* < 0.05; ^▲▲^*P* < 0.01, the model group vs. the curcumin (300 mg/kg) group.

To study the effect of curcumin on renal pathology in PHN rats, the glomerular and tubular structures were assessed by HE, PAS, and Masson staining, respectively. The HE staining revealed that the glomerular capillary lumen was uniform and consistent, the basement membrane of epithelial cells was intact, and no inflammatory cell infiltration was observed in the renal interstitium of the control group. However, there was glomerular hypertrophy and deformity, tubular lumen dilatation, inflammatory cell infiltration, and basement membranes thickened in rats of the model group. After treatment with curcumin, the expansion of glomeruli was significantly decreased, and the inflammatory cell infiltration was markedly reduced (Figure 1(a)). The representative photomicrographs of rat kidney in PAS-stained showed that the cell nucleus was blue and the glomerular basement membrane was purplish-red, and curcumin treatment significantly reduced the content of PAS-positive area in the curcumin group (Figures 1(a) and 1(b)). Previous studies indicated that tubulointerstitial fibrosis is the primary histopathological process of renal diseases. As shown in Figure 1(a), the blue area represented the degree of renal fibrosis in the Masson staining. As expected, the blue-positive area of the kidney of the PHN rats was significantly reduced in the curcumin group (Figures 1(a) and 1(c)). Thus, curcumin improves the histopathology changes of the kidneys in PHN rats.

### 3.3. Effects of Curcumin on Podocyte Loss in PHN Rats

**Figure 2:**
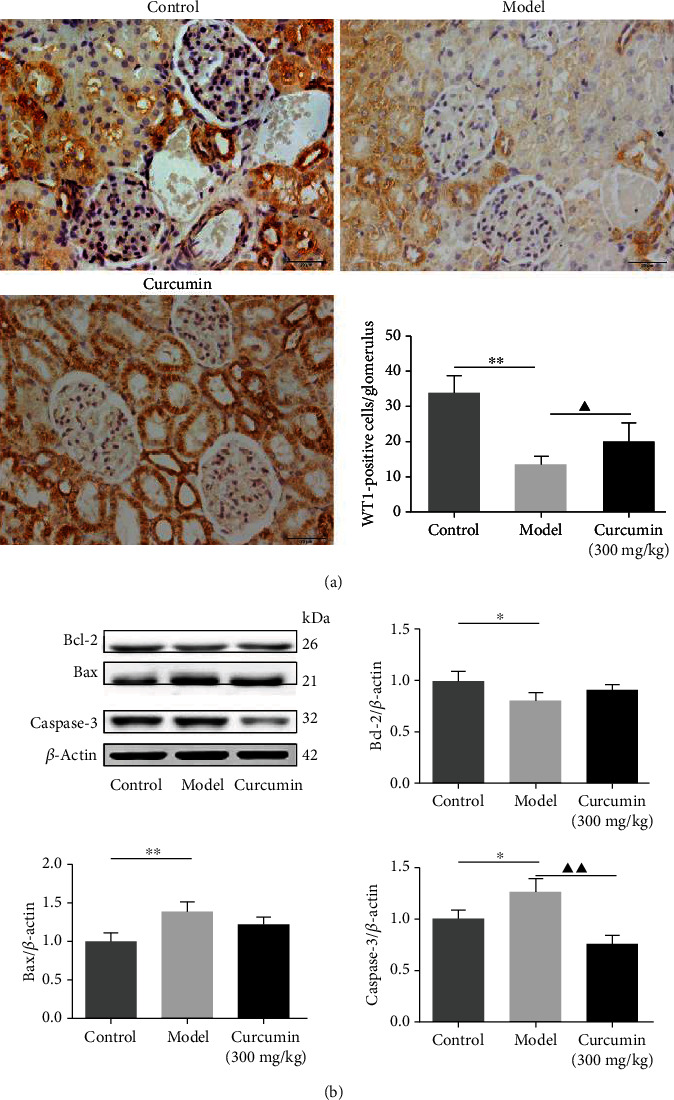
Curcumin attenuated the podocyte loss and apoptosis in PHN rats. (a) Podocyte number in the glomerular was estimated with the immunohistochemistry (scale bars 20 *μ*m, designated by the percentage of positive areas in glomerulus). (b) Expression of Bcl-2, Bax, and Caspase-3, the apoptosis-related proteins in renal tissues. ^∗^*P* < 0.05; ^∗∗^*P* < 0.01, the model group vs. the control group, ^▲^*P* < 0.05; ^▲▲^*P* < 0.01, the model group vs. the curcumin (300 mg/kg) group.

To further evaluate the effects of curcumin in the kidney of PHN rats, we next investigate the glomerular podocyte number by using immunohistochemical staining. As shown in Figure 2(a) and supplementary file Figure S1, the number of the glomerular podocyte, as presented as WT1-positive cells, was significantly decreased in the model group compared with the control group. Encouragingly, curcumin administration significantly reduced the podocyte loss in the glomeruli of the curcumin group compared with that in the model group. Furthermore, to explore whether the loss of podocyte resulted from the cell apoptosis in the kidney, the total protein expression of Bcl-2, Bax, and Caspase-3 was measured by western blotting. The results demonstrated that the Bcl-2 level in the curcumin group presented an increasing trend, and the Bax expression in the curcumin group showed a decreasing trend, while the difference was not significant. Besides, under the treatment of curcumin, which caused a significant elimination in the protein expression level of Caspase-3 compared with the model group (Figure 2(b)), these findings suggested that curcumin treatment could effectively improve podocyte loss and inhibit the nephritic apoptosis level in PHN rats.

### 3.4. Effects of Curcumin on Autophagy in PHN Rats

**Figure 3:**
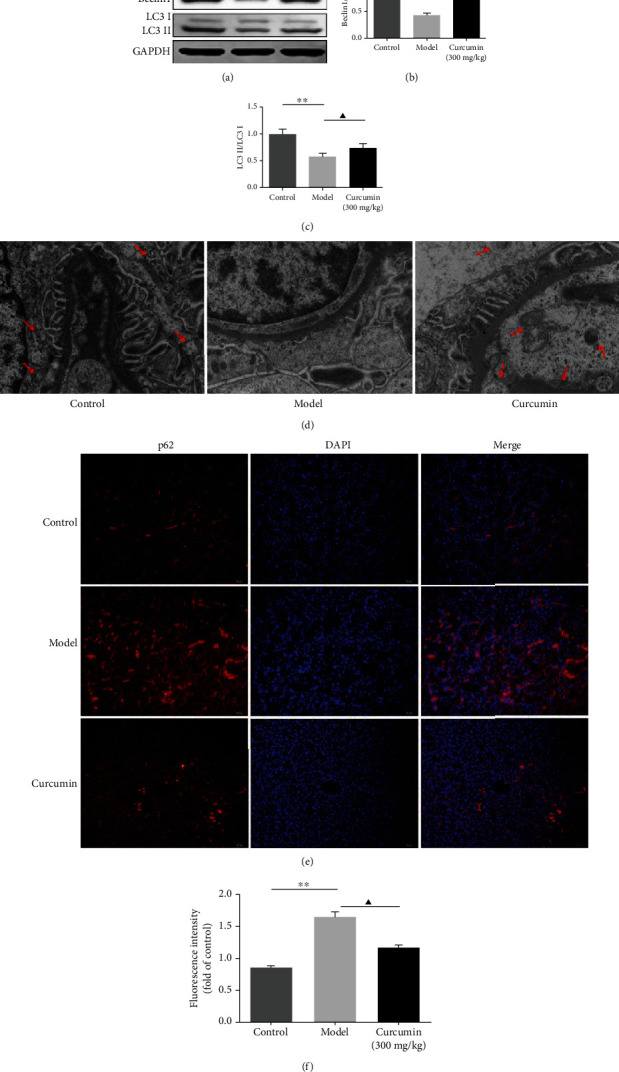
Curcumin treatment improves the autophagy of podocyte in PHN rats. (a) Representative western blotting images of beclin1, LC3 II, and LC3 I in the renal tissues of PHN rats. GAPDH is used as a loading control. (b) Quantification of beclin1 in the PHN rats. (c) Quantification of the LC3 II/LC3 I ratio in the PHN rats. (d) The ultrastructure of autophagosomes (*arrows*) of podocytes in the control and model groups was detected by the transmission electron microscopy (TEM, original magnification ×6000). (e) Immunofluorescence staining for p62 expression in the renal tissues of PHN rats (scale bars 20 *μ*m). (f) Analysis of fluorescence intensity of p62-positive cells in the PHN rats. ^∗∗^*P* < 0.01, the model group vs. the control group, ^▲^*P* < 0.05 the model group vs. the curcumin (300 mg/kg) group.

Compared with the control group, western bolting results illustrated the levels of beclin1 and the LC3 II/I ratio was remarkably decreased in the model group, while treatment with curcumin markedly upregulated the expression level of beclin1, and the LC3 II/I ratio in the curcumin-treated group (Figures 3(a)–3(c) and supplementary file Figure S2). Additionally, the results from TEM showed that, within the control group, the glomerular podocytes were uniformly distributed and distinct with multiple autophagic vacuoles; in the model group, the structure of glomerular podocyte became fuzzy, and the podocyte foot processes were significantly reduced and showed different degrees of fusion; autophagic vacuoles were not present or disappeared (Figure 3(d)). Meanwhile, compared with the model group, the fusion of podocyte foot processes was significantly ameliorated, and autophagic vacuoles were observed in the curcumin group. Also, Immunofluorescence staining assay indicated the expression of the autophagy marker p62. Curcumin significantly decreased the expression level of p62 in the treated group compared with that in the model group (Figures 3(e) and 3(f)). Therefore, curcumin increased the autophagy in renal tissues of PHN rats.

### 3.5. Effects of Curcumin on PI3K/AKT/mTOR and Nrf2/HO-1 in Renal Tissues of PHN Rats

**Figure 4:**
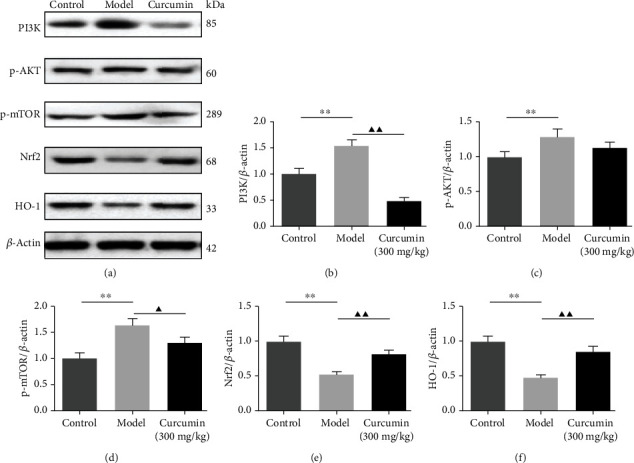
Curcumin blocks the PI3K/AKT/mTOR pathway and activates the Nrf2/HO-1 pathway in the renal tissues of PHN rats. (a) Western blot image of PI3K, p-AKT, p-mTOR, Nrf2, and HO-1. *β*-Actin is selected as a loading control. (b–f) The statistical data of PI3K, p-AKT, p-mTOR, Nrf2, and HO-1 was analyzed with ImageJ v1.8.0 software. Data were expressed as mean ± SD, ^∗∗^*P* < 0.01, the model group vs. the control group, ^▲^*P* < 0.05; ^▲▲^*P* < 0.01, the model group vs. the curcumin (300 mg/kg) group.

To investigate the potential mechanism associated with the activation of autophagy and antioxidant stress properties of curcumin, the PI3K/AKT/mTOR and Nrf2/HO-1 pathway-related target expression levels were determined by western blot. The current results revealed that in the model group, the protein levels of PI3K, the phosphorylation level of AKT, and mTOR in kidney tissues of PHN rats were significantly increased (Figures 4(a)–4(d) and supplementary file Figure S3), while the protein expressions of Nrf2 and HO-1 were lower than that in the control (Figures 4(a), 4(e), and 4(f)). Interestingly, curcumin treatment could reverse the levels of PI3K/AKT/mTOR and Nrf2/HO-1 pathways to some extent in PHN rats.

## 4. Discussion

Membranous nephropathy (MN), an autoimmune disease, is one of the frequent causes of end-stage renal disease in adults. A recent retrospective study has suggested that the incidence of IMN is increasing, especially in the younger ones [33]. Its therapeutic regimen also is unsatisfactory and controversial due to the heterogeneity of the disease, although approximately a third of patients will achieve spontaneous remission [34]. There is an urgent need for more promising pharmaceuticals to promote treatment avenues. Curcumin has been used as a coloring agent, food additive owing to its anti-inflammatory, antioxidant, and also shown therapeutic activities in different kinds of diseases, including type 2 diabetes mellitus [35], Alzheimer's disease [36], and diabetic nephropathy [37]; however, the underlying mechanism of curcumin in MN remains unclear. In the current study, by establishing anti-Fx1A serum-induced experimental MN, we preliminarily investigated the renoprotective effects of curcumin on passive Heymann nephritis (PHN) rats and the potential mechanisms.

MN is characterized by the presence of heterologous IgG subepithelial immune complex deposits between the GBM and the podocyte, thus leading to heavy proteinuria during the course [38, 39]. The autologous stage of PHN mimics IMN, which is similar to the pathophysiological mechanisms of IMN in humans [39], and the PHN, a typical animal model of human MN, has been commonly used since first described by Heymann et al. in 1959 [40]. In this study, we found that curcumin at 300 mg/kg/d is effectively against renal function injury and reduced the levels of SCr, BUN, and urine albumin, as well as the podocyte loss. Also, pathological changes were attenuated in the curcumin-treated PHN rats; in addition, administration of curcumin was found to significantly alleviate oxidative stress. On the other hand, curcumin attenuated the apoptosis level in kidney tissues by upregulating the Bcl-2 expression and decreasing the protein expression levels of Bax and Caspase-3. These results are consistent with those reported in the literature [20], indicating that the administration of curcumin can improve renal function in PHN rats.

Podocytes are inherent terminally differentiated cells in the glomerulus with regularly spaced foot processes and play a crucial role in maintaining the integrity of the glomerular filtration barrier. Fan et al. also showed that podocyte damage can lead to an extensive fusion of foot processes and severe proteinuria in doxorubicin-induced nephrotic syndrome rats [41]. To further observe the effect of curcumin on podocyte injury, we performed a transmission electron microscopy test. Electron microscopic observation showed the extensive fusion of podocyte foot processes in the model rats. It has been documented that autophagy acts as a programmed cell survival process for recycling cytoplasmic macromolecules and organelles in cells via the lysosomal system to maintain the cell homeostasis. Additionally, on electron microscopy, the podocyte in the model rats revealed lacking autophagic vacuoles. In our experiments, the autophagic vacuoles and the active marker of autophagosomes, beclin1, and LC3 II/LC3 I decreased dramatically in PHN rats but increased after treatment with curcumin. These results revealed that the protective role of curcumin might be associated with the activation of autophagy in the renal tissue.

Emerging evidence has verified that the PI3K/Akt/mTOR pathway is an important intracellular signaling pathway that is related to extensive physiological events, including cell proliferation, migration, survival, and the regulation of autophagy of podocyte. A previous study has reported that curcumin exerts neuroprotective effects in cerebral ischemia-reperfusion by attenuating autophagic activities through mediating the PI3K/Akt/mTOR pathway [42]. However, the PI3K/Akt/mTOR pathway has not been investigated in the treatment of the experimental MN with curcumin. mTOR, a serine/threonine kinase, is regulating the cell growth and inhibited mTOR activity to strengthen autophagy in diabetic nephropathy [43]. Additionally, Li et al. observed that blocking the PI3K/Akt/mTOR pathway contributed to restoring the podocytic adhesive capacity damage and autophagy activity [44]. In our present study, curcumin treatment reduced the levels of PI3K, phosphorylated forms of Akt, and mTOR but increased the ratio of LC3 II/LC3 I, thus enhancing autophagy. In addition, several reports illustrated that oxidative stress has resulted from decreasing antioxidant capacity. In the present study, curcumin can effectively reduce MDA content and increase SOD, GSH, and CAT activity, suggesting the protective effect of curcumin on PHN rats associated with its antioxidant property. Therefore, we further investigate the antioxidant mechanism of curcumin. Besides, a recent study demonstrated the important role of the nuclear factor E2-related factor 2/heme oxygenase-1 (Nrf2/HO-1) pathway in the activation of autophagy under oxidative stress [45]. And our results indicate that the expression of the nuclear transcription factor Nrf2 and the antioxidant enzymes HO-1 is increased observably in the curcumin-treated group. The results of the present study suggested that the renoprotective effects of curcumin in MN could benefit from the modulation of autophagy through PI3K/Akt/mTOR and Nrf2/HO-1 pathways. However, several limitations of this study should be noted. First, further studies based on immunofluorescent or immunohistochemical staining are needed to explore the effect of curcumin on the subepithelial immune complex in PHN rats. Second, the effects of curcumin on autophagy-related mechanisms in cell trials still need to be further explored.

## 5. Conclusions

In summary, the current study demonstrates that curcumin has a promising therapeutic effect on MN by improving the kidney function of PHN rats, inhibiting kidney oxidative stress, and ameliorating podocyte injury, which is associated with the inhibition of PI3K/Akt/mTOR, and the strengthening of Nrf2/HO-1 signaling pathways. These results suggested that curcumin is a promising therapeutic strategy in MN treatment. However, further clinical trials are required to confirm the therapeutic rationale of these findings.

## Figures and Tables

**Table 1:** Effect of curcumin treated on biochemical parameters of PHN rats.

Biologic samples	Parameter	Control	Model	Curcumin (300 mg/kg)
Serum	ALB (g·L^−1^)	42.08 ± 1.85^∗∗^	35.64 ± 1.04	40.67 ± 1.25^▲^
	TP (g·L^−1^)	58.64 ± 1.32^∗∗^	45.36 ± 1.46	56.67 ± 1.78^▲▲^
	TC (mmol·L^−1^)	1.9 ± 0.33^∗∗^	5.76 ± 0.87	3.14 ± 0.64^▲^
	TG (mmol·L^−1^)	0.22 ± 0.07^∗∗^	0.92 ± 0.16	0.33 ± 0.18^▲▲^
	SCr (*μ*mol/L)	37.19 ± 5.36^∗∗^	82.45 ± 10.03	53.46 ± 7.13^▲^
	BUN (mmol·L^−1^)	8.37 ± 1.22^∗∗^	17.56 ± 1.68	14.36 ± 1.17^▲▲^

Urine	Urine volume (mL)	21.30 ± 3.47^∗∗^	42.00 ± 1.28	29.64 ± 1.16^▲▲^
	Urine albumin (mg/24 h)	10.35 ± 1.36^∗∗^	68.46 ± 7.64	31.87 ± 3.74^▲▲^

Notes: the control group vs. the model group ^∗∗^*P* < 0.01; the curcumin group vs. the model group ^▲^*P* < 0.05; ^▲▲^*P* < 0.01.

**Table 2:** Effect of curcumin treated on the oxidative stress level of kidney tissues in PHN rats.

Homogenate	Parameter	Control	Model	Curcumin (300 mg/kg)
Kidney	SOD (U·mg^−1^)	73.86 ± 0.57^∗∗^	34.36 ± 0.69	51.54 ± 0.74^▲▲^
	GSH (U·mg^−1^)	155.02 ± 2.75^∗∗^	64.64 ± 2.07	92.67 ± 3.23^▲▲^
	CAT (U·mg^−1^)	3.8 ± 0.93^∗∗^	1.76 ± 0.85	2.64 ± 0.65^▲^
	MDA (mmol·mg^−1^)	1.42 ± 0.08^∗∗^	9.57 ± 0.54	2.67 ± 0.52^▲▲^

Notes: the control group vs. the model group ^∗∗^*P* < 0.01; the curcumin group vs. the model group ^▲^*P* < 0.05; ^▲▲^*P* < 0.01.

## Data Availability

The analyzed data of this study are available from the corresponding author on reasonable request.

## Supplementary Materials

Supplementary 1 Supplementary File Figure S1: full unedited versions of the western blots for Figure 2(b).

Supplementary 2 Supplementary File Figure S2: full unedited versions of the western blots for Figure 3(a).

Supplementary 3 Supplementary File Figure S3: full unedited versions of the western blots for Figure 4(a).
