# Supplementary material for: Curcumin Improves the Renal Autophagy in Rat Experimental Membranous Nephropathy via Regulating the PI3K/AKT/mTOR and Nrf2/HO-1 Signaling Pathways
Source: Biomed Res Int. 2020 Nov 1;2020:7069052. doi: 10.1155/2020/7069052 (PMC7654212; doi:10.1155/2020/7069052)
Supplement: Supplementary 1 — Supplementary File Figure S1: full unedited versions of the western blots for Figure 2(b). [file 7069052.f1.pdf]

supplementary file Figure S1

Full unedited versions of the western blots for Figure 2B

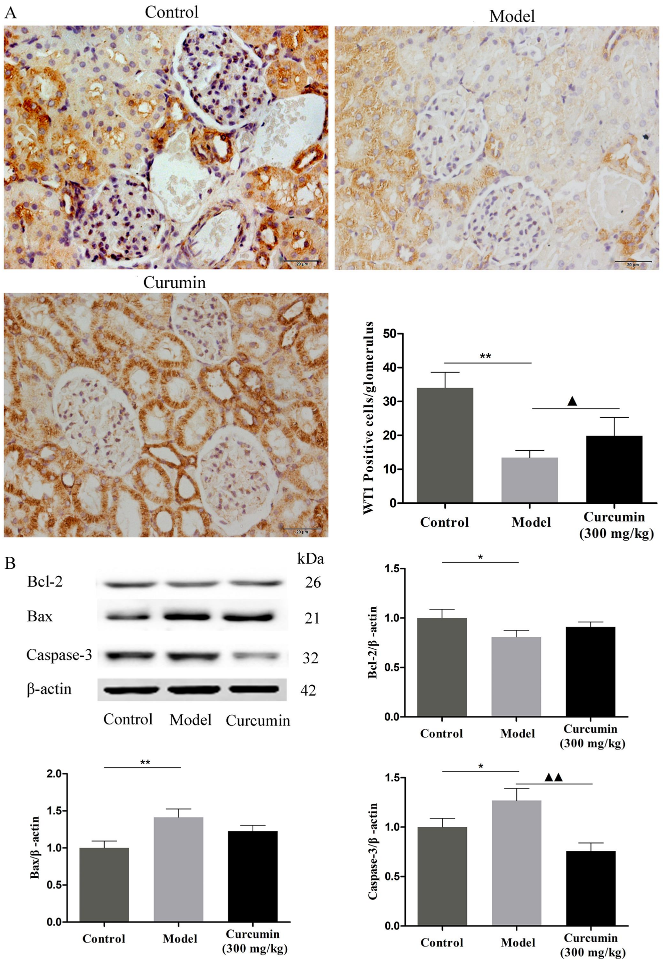

Figure 2 Curcumin attenuated the podocyte loss and apoptosis in PHN rats. A Podocyte number in the glomerular was estimated with the immunohistochemistry (Scale bars 20 μm). **B** Expression of Bcl-2, Bax, and Caspase-3, the apoptosis-related proteins in renal tissues. **P<0.05*; ***P<0.01*, the model group vs the control group, ▲*P<0.05*; ▲▲ *P<0.01*, the model group vs the curcumin (300 mg/kg) group.

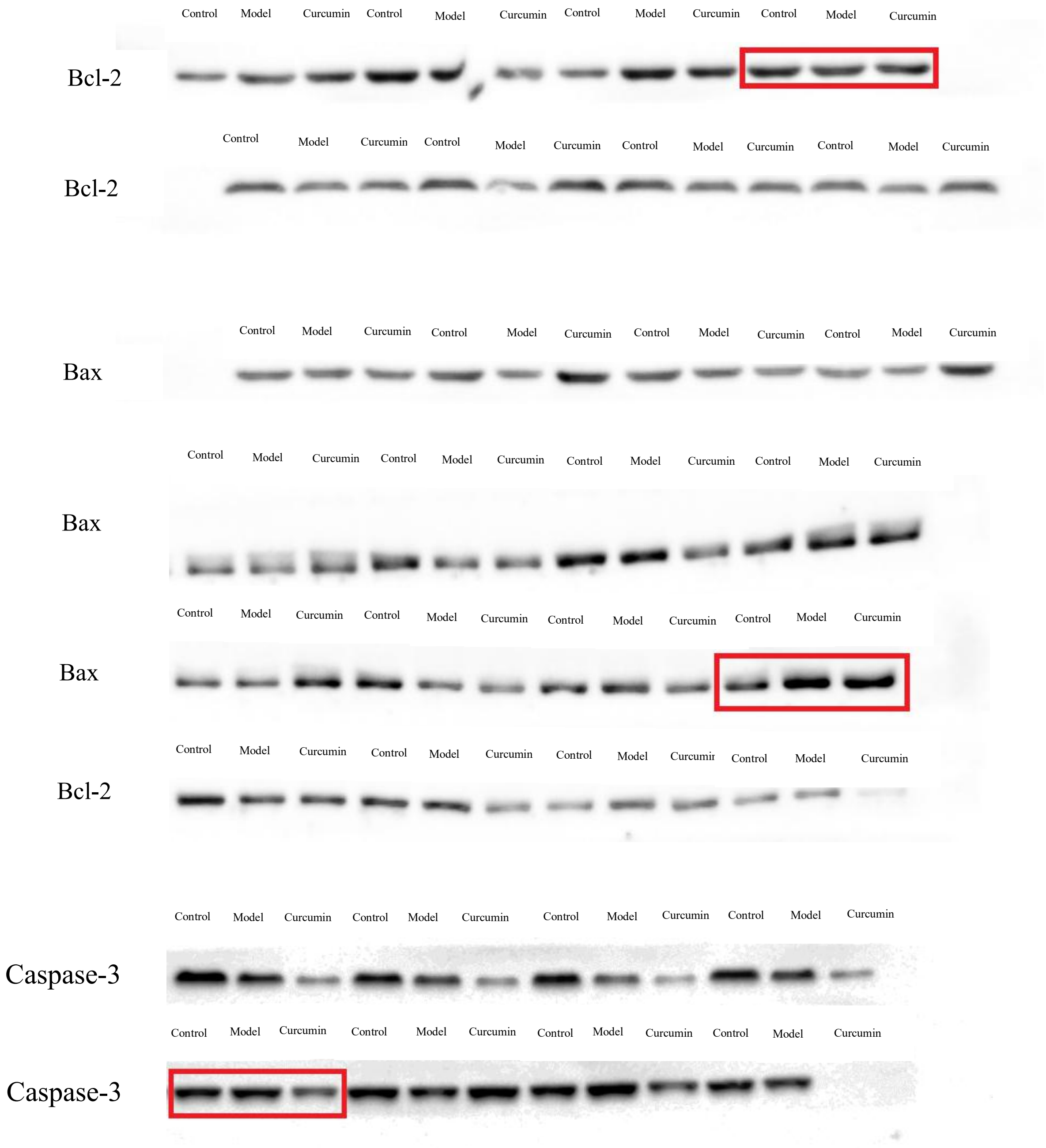
Control Model Curcumin Control Model Curcumin Control Model Curcumin Control Model Curcumin
Bcl-2
Control Model Curcumin Control Model Curcumin Control Model Curcumin Control Model Curcumin
Bcl-2
Control Model Curcumin Control Model Curcumin Control Model Curcumin Control Model Curcumin
Bax
Control Model Curcumin Control Model Curcumin Control Model Curcumin Control Model Curcumin
Bax
Control Model Curcumin Control Model Curcumin Control Model Curcumin Control Model Curcumin
Bax
Control Model Curcumin Control Model Curcumin Control Model Curcumir Control Model Curcumin
Bcl-2
Control Model Curcumin Control Model Curcumin Control Model Curcumin Control Model Curcumin
Caspase-3
Control Model Curcumin Control Model Curcumin Control Model Curcumin Control Model Curcumin
Caspase-3

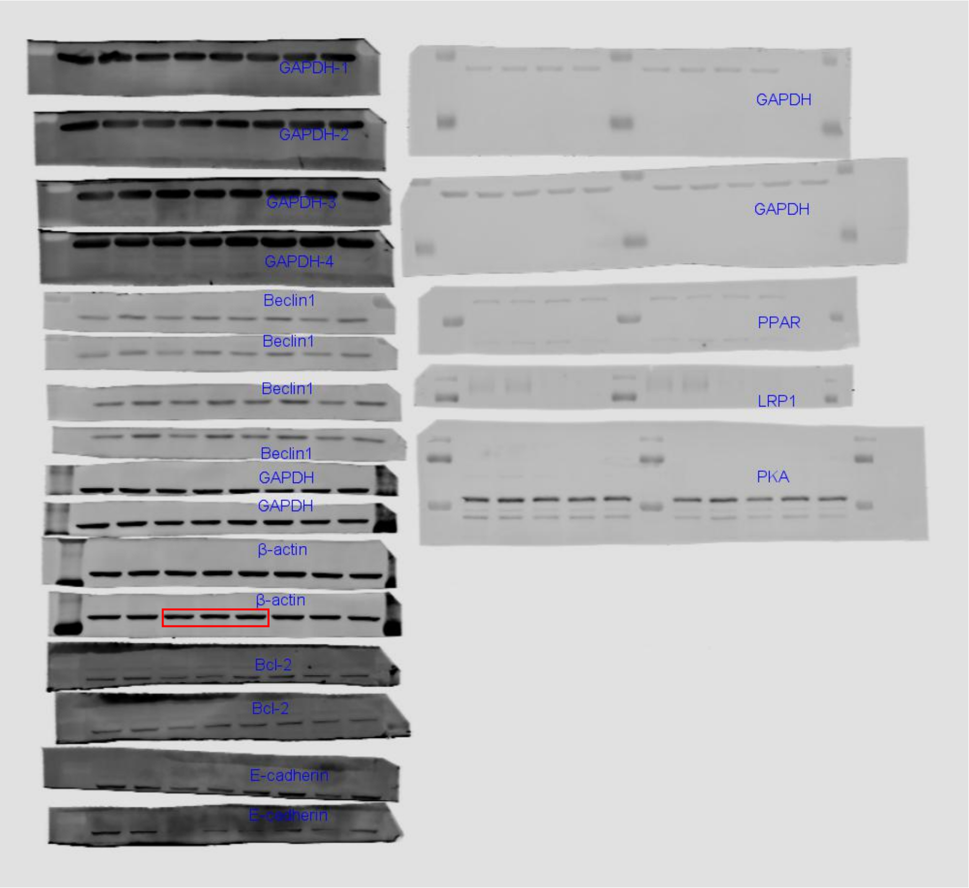
GAPDH-1
GAPDH-2
GAPDH-3
GAPDH-4
Beclin1
Beclin1
Beclin1
Beclin1
GAPDH
GAPDH
β-actin
β-actin
Bcl-2
Bcl-2
E-cadherin
E-cadherin
GAPDH
GAPDH
PPAR
LRP1
PKA
