# Supplementary material for: Curcumin Improves the Renal Autophagy in Rat Experimental Membranous Nephropathy via Regulating the PI3K/AKT/mTOR and Nrf2/HO-1 Signaling Pathways
Source: Biomed Res Int. 2020 Nov 1;2020:7069052. doi: 10.1155/2020/7069052 (PMC7654212; doi:10.1155/2020/7069052)
Supplement: Supplementary 2 — Supplementary File Figure S2: full unedited versions of the western blots for Figure 3(a). [file 7069052.f2.pdf]

supplementary file Figure S2

Full unedited versions of the western blots for Figure 3A

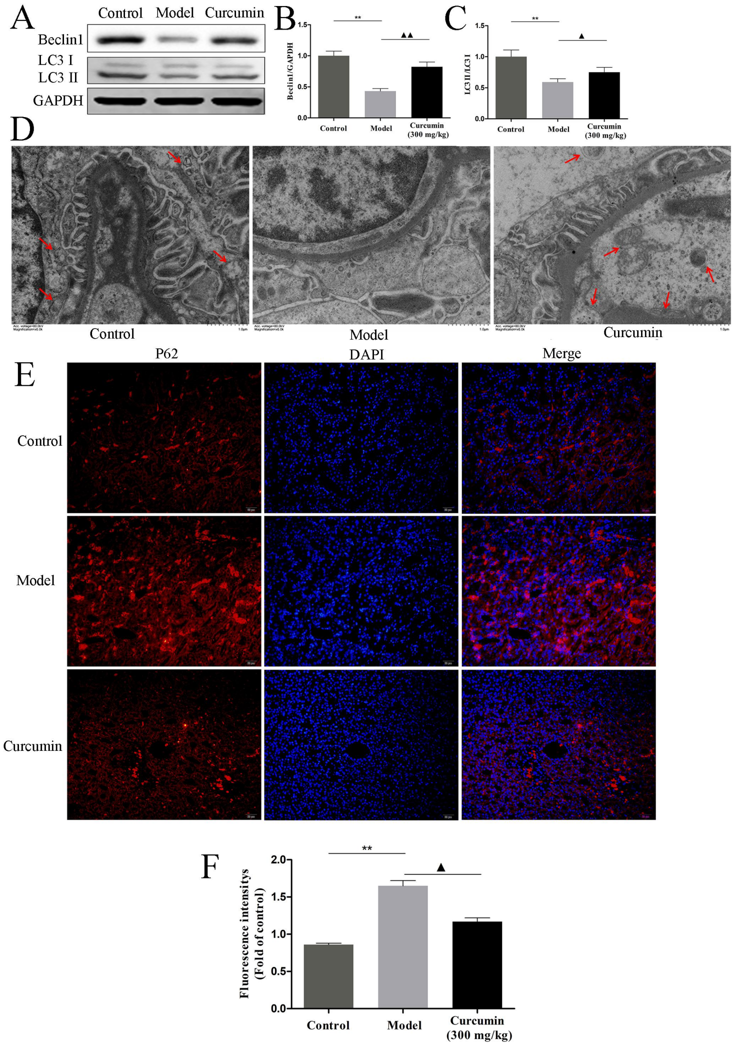

Figure 3 Curcumin treatment improves the autophagy of podocyte in PHN rats. A Representative Western blotting images of beclin1, LC3-II, and LC3-I in the renal tissues of PHN rats. GAPDH is used as a loading control. **B** Quantification of beclin1 in the PHN rats. **C** Quantification of the LC3-II/LC3-I ratio in the PHN rats. **D** Ultrastructure of autophagosomes (*arrows*) of podocytes in the control and model group were detected by the transmission electron microscopy (TEM, original magnification ×6000) **E** Immunofluorescence staining for p62 expression in the renal tissues of PHN rats (Scale

bars 20 μm). **F** Analysis of fluorescence intensity of p62 positive cells in the PHN rats. ***P<0.01*, the model group vs the control group, $^{\blacktriangle}$*P<0.05* the model group vs the curcumin (300 mg/kg) group.

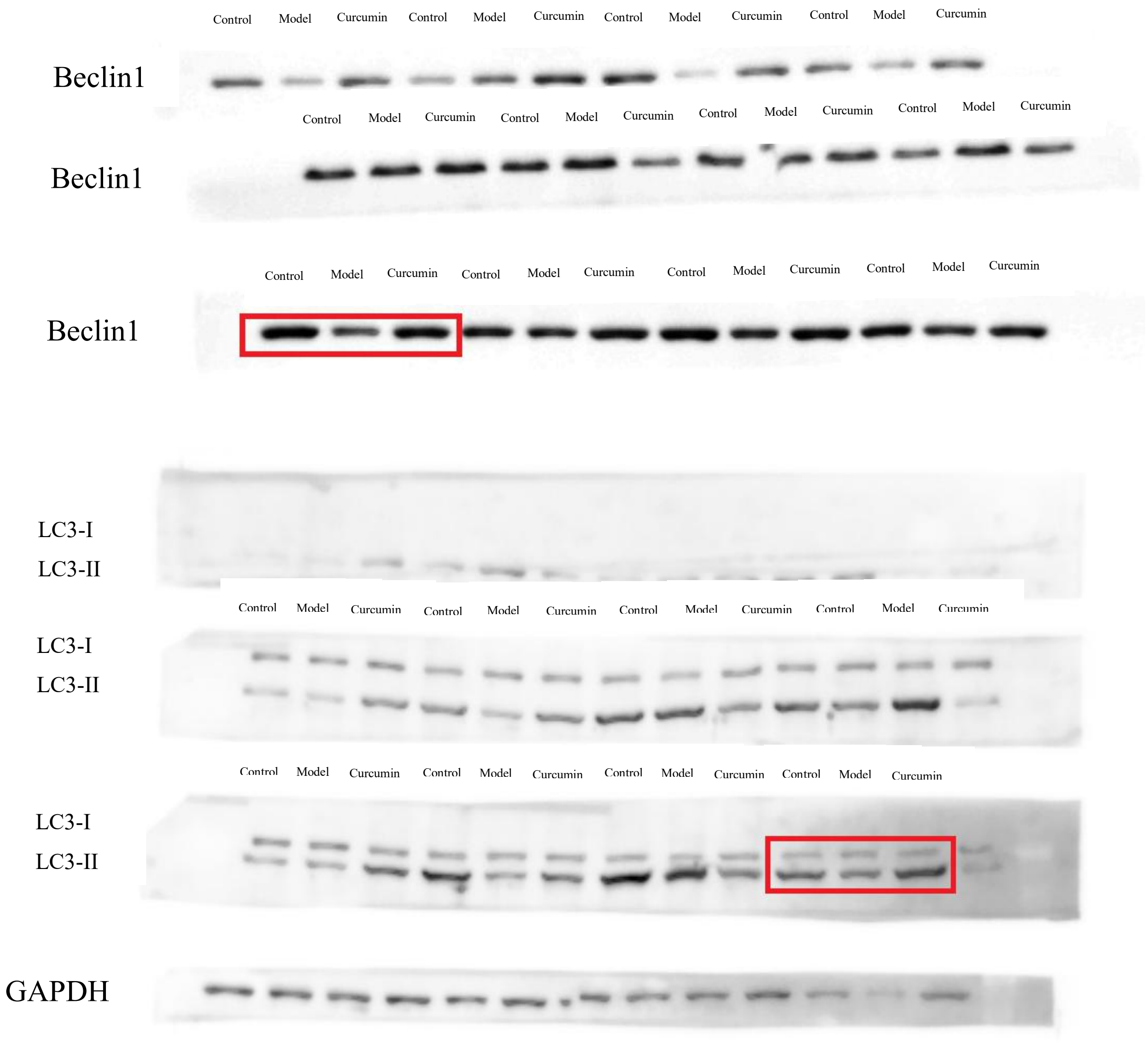

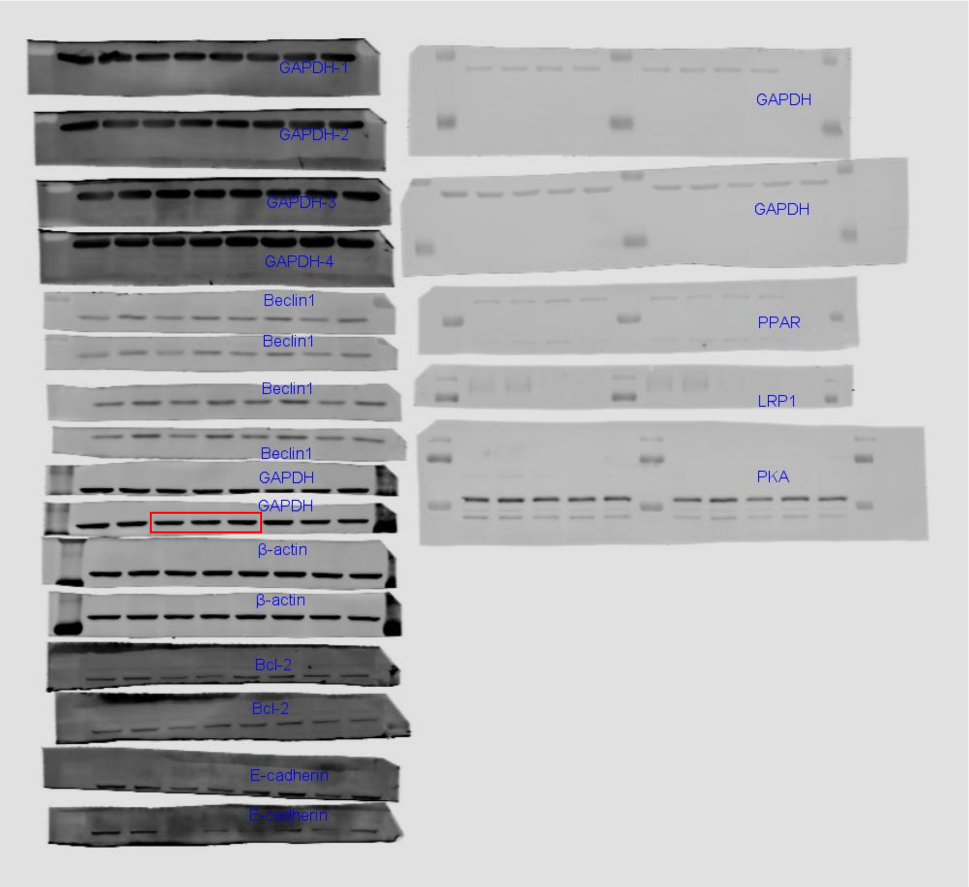
GAPDH-1
GAPDH-2
GAPDH-3
GAPDH-4
Beclin1
Beclin1
Beclin1
Beclin1
GAPDH
GAPDH
β-actin
β-actin
Bcl-2
Bcl-2
E-cadherin
E-cadherin
GAPDH
GAPDH
PPAR
LRP1
PKA
