# Supplementary material for: Curcumin Improves the Renal Autophagy in Rat Experimental Membranous Nephropathy via Regulating the PI3K/AKT/mTOR and Nrf2/HO-1 Signaling Pathways
Source: Biomed Res Int. 2020 Nov 1;2020:7069052. doi: 10.1155/2020/7069052 (PMC7654212; doi:10.1155/2020/7069052)
Supplement: Supplementary 3 — Supplementary File Figure S3: full unedited versions of the western blots for Figure 4(a). [file 7069052.f3.pdf]

supplementary file Figure S3

Full unedited versions of the western blots for Figure 4A

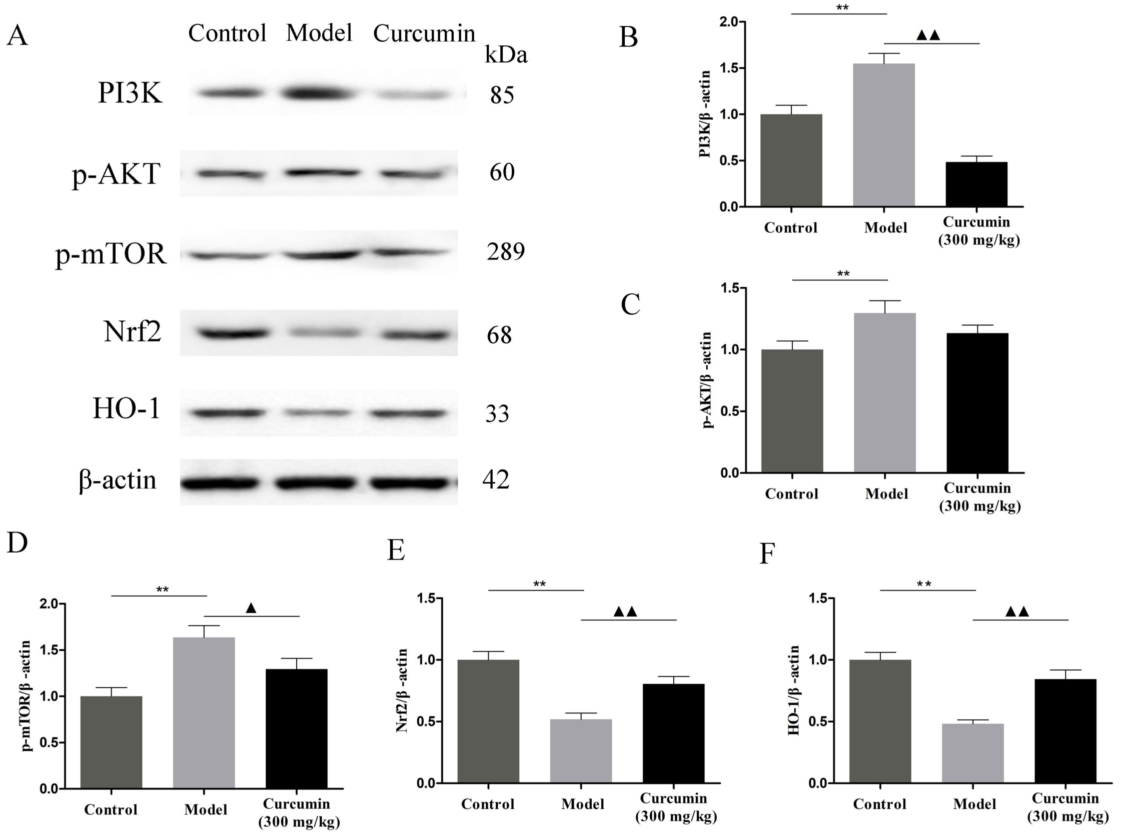

Figure 4 Curcumin blocks PI3K/AKT/mTOR pathway and activates the Nrf2/HO-1 pathway in the renal tissues of PHN rats. A Western blot image of PI3K, p-AKT, p-mTOR, Nrf2, and HO-1. β-actin is selected as a loading control. **B-F** The statistical data of PI3K, p-AKT, p-mTOR, Nrf2, and HO-1 was analyzed with Image J v1.8.0 software. Data were expressed as mean ± SD, ***P<0.01*, the model group vs the control group, $^{▲}$*P<0.05*; $^{▲▲}$*P<0.01*, the model group vs the curcumin (300 mg/kg) group.

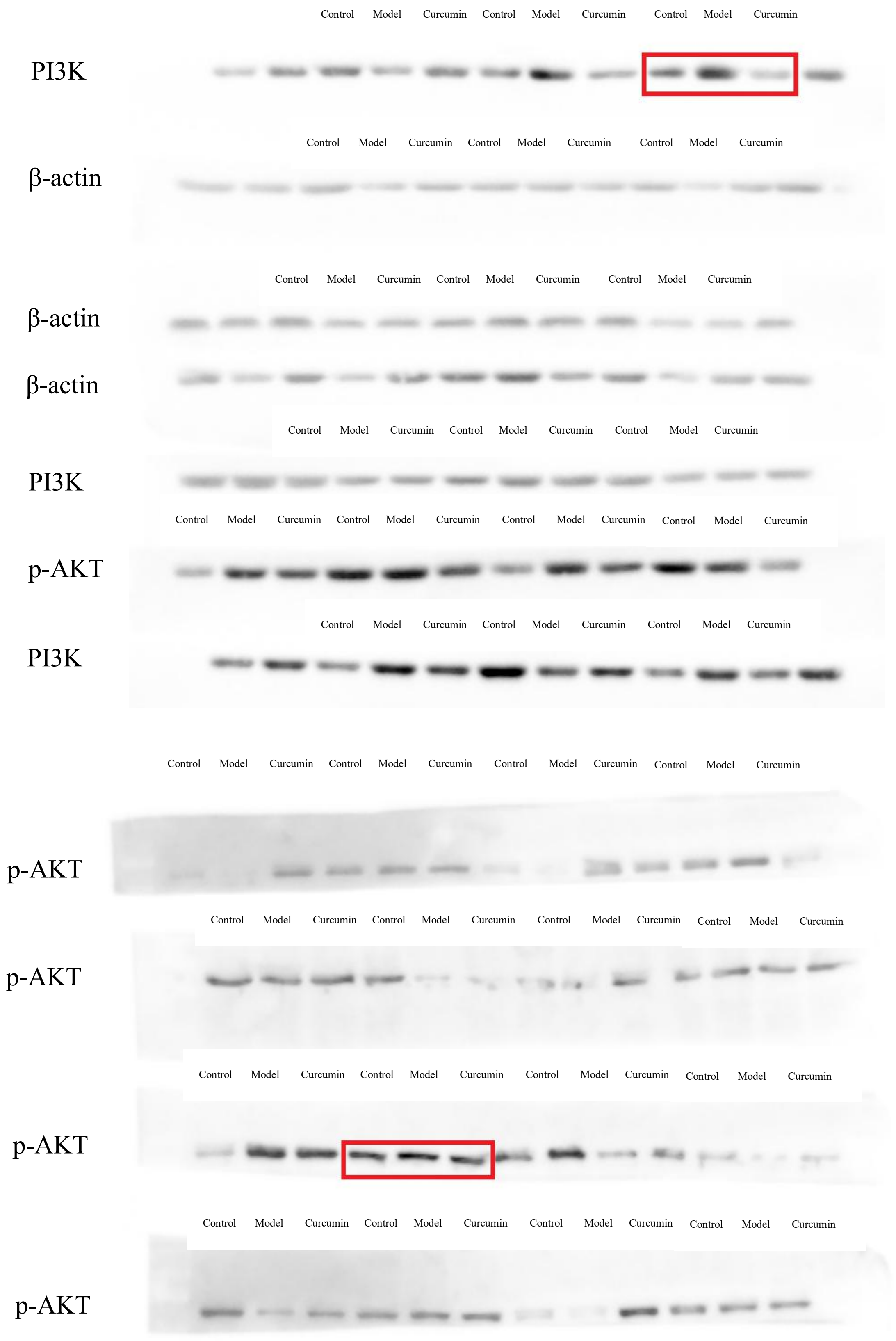
Control Model Curcumin Control Model Curcumin Control Model Curcumin
PI3K
Control Model Curcumin Control Model Curcumin Control Model Curcumin
β-actin
Control Model Curcumin Control Model Curcumin Control Model Curcumin
β-actin
β-actin
Control Model Curcumin Control Model Curcumin Control Model Curcumin
PI3K
Control Model Curcumin Control Model Curcumin Control Model Curcumin Control Model Curcumin
p-AKT
Control Model Curcumin Control Model Curcumin Control Model Curcumin
PI3K
Control Model Curcumin Control Model Curcumin Control Model Curcumin Control Model Curcumin
p-AKT
Control Model Curcumin Control Model Curcumin Control Model Curcumin Control Model Curcumin
p-AKT
Control Model Curcumin Control Model Curcumin Control Model Curcumin Control Model Curcumin
p-AKT
Control Model Curcumin Control Model Curcumin Control Model Curcumin Control Model Curcumin
p-AKT

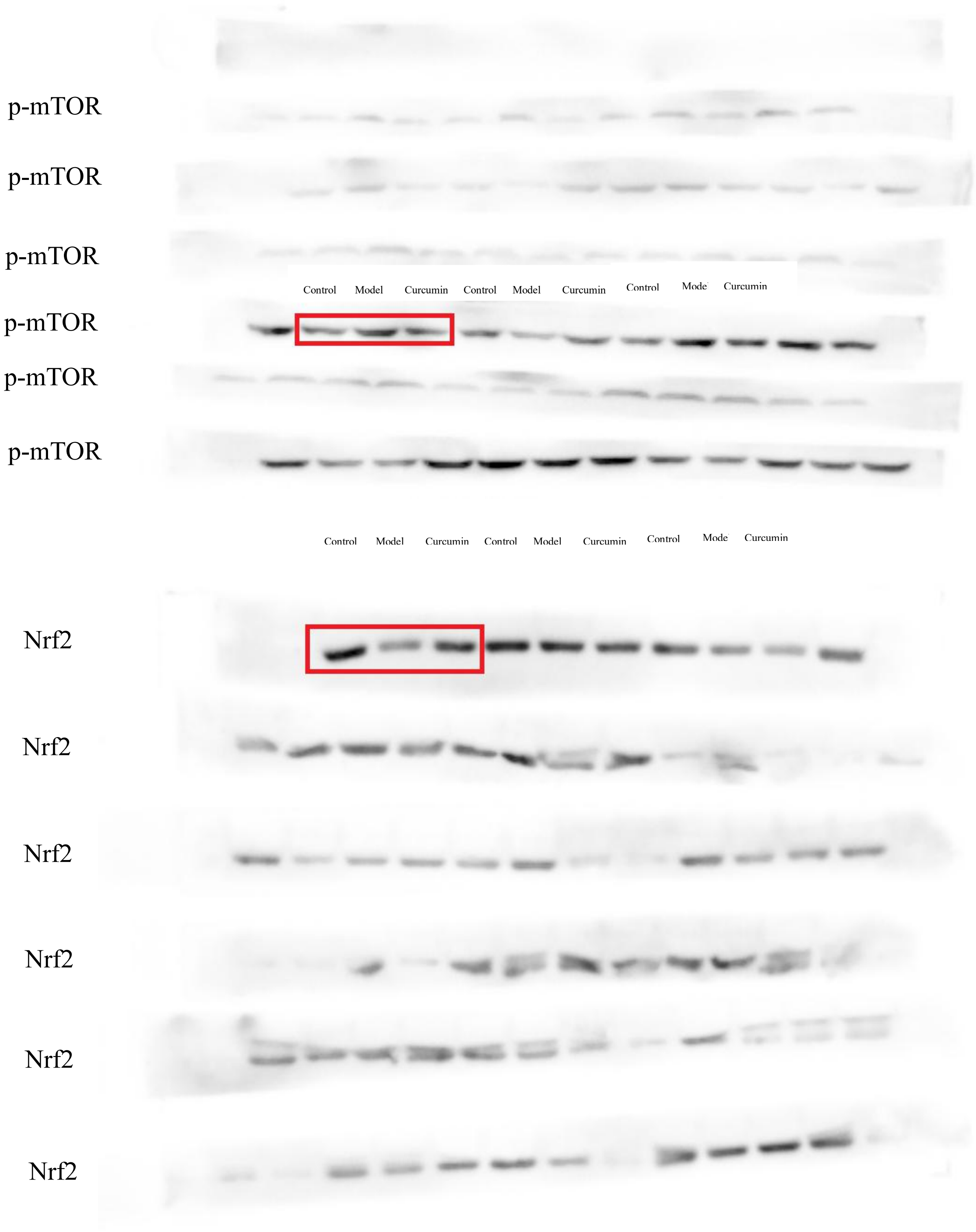
p-mTOR
p-mTOR
p-mTOR
Control Model Curcumin Control Model Curcumin Control Mode Curcumin
p-mTOR
p-mTOR
p-mTOR
Control Model Curcumin Control Model Curcumin Control Mode Curcumin
Nrf2
Nrf2
Nrf2
Nrf2
Nrf2
Nrf2

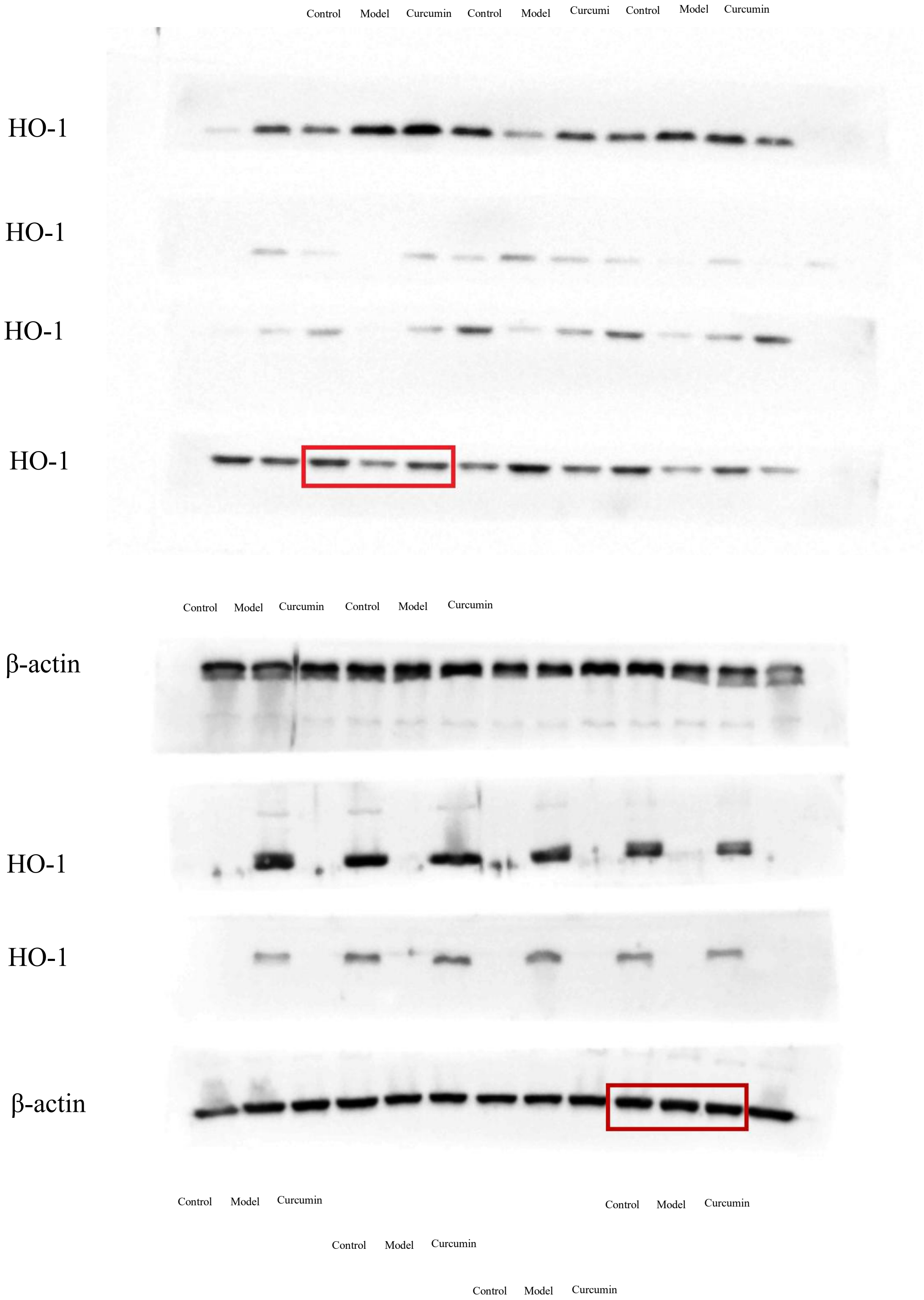
Control Model Curcumin Control Model Curcumi Control Model Curcumin
HO-1
HO-1
HO-1
HO-1
Control Model Curcumin Control Model Curcumin
β-actin
HO-1
HO-1
β-actin
Control Model Curcumin
Control Model Curcumin
Control Model Curcumin
Control Model Curcumin
